# Online Artifact Removal for Brain-Computer Interfaces Using Support Vector Machines and Blind Source Separation

**DOI:** 10.1155/2007/82069

**Published:** 2007-11-13

**Authors:** Sebastian Halder, Michael Bensch, Jürgen Mellinger, Martin Bogdan, Andrea Kübler, Niels Birbaumer, Wolfgang Rosenstiel

**Affiliations:** ^1^Institute of Medical Psychology and Behavioral Neurobiology, University of Tübingen, Gartenstr. 29, 72074 Tübingen, Germany; ^2^Wilhelm-Schickard Institute for Computer Engineering, University of Tübingen, Sand 13, 72076 Tübingen, Germany; ^3^Computer Engineering, Institute of Computer Science, Faculty of Mathematics and Computer Science, University of Leipzig, Johannisgasse 26, 04103 Leipzig, Germany; ^4^National Institutes of Health (NIH), National Institute of Neurological Disorders and Stroke (NINDS), Human Cortical Physiology Section, Bethesda, MD 20892, USA

## Abstract

We propose a combination of blind source separation (BSS) and independent component analysis (ICA) (signal decomposition into artifacts and nonartifacts) with support vector machines (SVMs) (automatic classification) that are designed for online usage. In order to select a suitable BSS/ICA method, three ICA algorithms (JADE, Infomax, and FastICA) and one BSS algorithm (AMUSE) are evaluated to determine their ability to isolate electromyographic (EMG) and electrooculographic (EOG) artifacts into individual components. An implementation of the selected BSS/ICA method with SVMs trained to classify EMG and EOG artifacts, which enables the usage of the method as a filter in measurements with online feedback, is described. This filter is evaluated on three BCI datasets as a proof-of-concept of the method.

## 1. INTRODUCTION

Since the discovery of the human electroencephalogram (EEG) activity in 1929 by Hans Berger [1], EEG measurements were mainly used for medical reasons or for research in the area of brain function. In the past 15 years, applications have been developed allowing the use of EEG activity as a nonmuscular communication channel or as an aid in motor restoration after paralysis, so-called brain-computer interfaces (BCIs) [2]. The idea is to provide completely paralyzed patients with a rudimentary communication channel by classifying the EEG signal (currently with information transfer rates between 10–25 bits/min [2]). Further progress of BCI systems depends on the development of new training methods for patients, the identification of signals best suited for voluntary control, and the removal of noise interfering with these signals.

Noise includes artifacts (we define any noncentral nervous system (CNS) signal recorded by the EEG to be an artifact) introduced either by the subject himself or by an external source. Artifacts in EEG recordings can be caused by eye blinks, eye movement, muscle and cardiac noise, as well as nonbiological sources (e.g., power-line noise). A problem arises if the artifacts generated by the subject are used to control the BCI system, because this violates the definition of a BCI as a nonmuscular communication channel. Furthermore, subjects with degenerative diseases would eventually lose this ability. For instance, these artifacts could be a voluntary or involuntary blinks or muscle contractions when the task is presented. Additionally, involuntary muscle or ocular activity might obscure the actual EEG signal, obstructing measurement of the features used to control the system. Electromyographic (EMG) activity tends to overlap EEG from 8 Hz upwards, whereas electrooculographic (EOG) activity overlaps in the range 0–12 Hz (e.g., the μ-rhythm has a frequency of 8–12 Hz). An overview of the effect of EMG on BCI training sessions is given in [3].

A supervisor can detect artifacts visually by analyzing the topographic and spectral properties of a signal. Unfortunately, the contaminated sections cannot just be rejected, due to the data loss this implies (blinks occur with a frequency of about 20 per minute and a duration between 50 and 500 milliseconds [4, 5]). Additionally, if the recordings are very long, the process of manual artifact rejection implies a significant increase in the time needed to fully process a dataset. Automating rejection would speed up this process but still cause the same amount of data loss. Therefore, the only feasible approach is to remove artifacts without affecting the remaining EEG data.

A comprehensive review of numerous artifact removal techniques and their application in BCI studies can be found in [6]. For example, a simple approach is highpass filtering the data to remove EOG and lowpass filtering the data to remove EMG artifacts, see, for example, [7]. This method will remove any brain signals of interest in the same frequency range. If a reliable reference channel is available, for example, an EOG recording, regression in the time, or frequency domain can be performed [8, 9]. The disadvantage of this method is that EOG recordings often contain brain signals which would inevitably also be removed. Nonetheless, it has been shown that this is preferable over artifact rejection methods because of the reduced amount of data loss [10].

An alternative approach is to use blind source separation (BSS), which is based on estimating the underlying sources (components) that compose the signals measured with the EEG. Ideally, the estimated components contain either artifacts or EEG activity. It is then possible to remove artifacts by elimination of the corresponding components. Makeig was one of the first to demonstrate the possibility of applying ICA methods to perform BSS on EEG data [11].

Artifacts were successfully isolated into a few output components and removed from the data. Advantages of using ICA to remove EOG artifacts from EEG data, instead of rejection- or regression-based techniques, have been shown in several studies, indicating that the percentage of brain signal that is removed with the EOG is reduced [12, 13]. EOG artifact removal on the basis of isolation into independent components (ICs) using BSS has been demonstrated in [14]. Another approach employs the second order statistics based “algorithm for multiple unknown signals extraction (AMUSE)” to detect EEG artifacts in sleep studies [15, 16].

In this paper, we propose an artifact removal method that removes artifacts whilst causing only minimal data loss and it is applicable in online environments and has no need for user interaction. This filter is implemented by using BSS/ICA algorithms in conjunction with support vector machine (SVM) classification and is based on an online capable design. The use of BSS/ICA algorithms minimizes data loss as the artifacts are isolated into ICs, automatic classification of these ICs with SVMs makes user interaction unnecessary, and the online capable design of the artifact filter provides a continuous stream of data in online settings. To make an objective selection of the algorithm being in the filter, possible, three ICA algorithms and one BSS algorithm are evaluated to determine their artifact isolation capabilities. An SVM is trained to classify artifacts on the basis of ICs extracted from data recorded for this purpose. Finally, we demonstrate the functionality of the filter using existing BCI data recorded from healthy and paralyzed participants.

## 2. METHODS

As a data model we assume a linear mixture of brain signals and artifact signals. This model corresponds to the model underlying BSS/ICA algorithms. As far as this model is accurate, artifacts can be removed from the EEG signal with an artifact removal matrix. This matrix is continuously updated and is calculated from SVM classification results on ICs determined with BSS/ICA.

A comparison to determine the best algorithm for the isolation of artifacts was conducted before training the SVMs. This comparison was limited to four algorithms which were selected because they cover the various principles of IC estimation employed in BSS algorithms. AMUSE [16] is a BSS method restricted to second-order statistics, JADE [17] a tensorial ICA method, Infomax [18] is based on maximum likelihood estimation, and FastICA [19] on the maximization of nongaussianity. After the selection of the best performing algorithms, SVMs were trained with artifact data recorded specifically for this purpose. A discussion of the filter design and the methods to achieve online functionality follows.

All of the BSS/ICA algorithms are contained in EEGLAB [20] and/or ICALAB [21].

### 2.1. BSS evaluation

#### 2.1.1. Method of BSS evaluation

The method that was used to evaluate the performance of the four BSS algorithms is discussed here. All algorithms were evaluated by mixing a known artifact component with an artifact-free background EEG signal, re-extracting the known component and measuring the correlation of the extracted components and the introduced artifact components, as done in [24].

EEG data recorded while the subject was performing no particular task was cleaned of blinks and other obvious artifacts by removing the corresponding sections using EEGLAB and then used as background $B_{\text{orig}}$. To obtain the first artifact source, an EMG recording was made on the forearm of a subject. This ensures that no CNS signals are contained in this artifact component. We assume that the spectral properties of an EMG signal generated at the forearm are comparable to those generated by muscles located on the head, for example, jaw muscles. This assumption seems to be a feasible tradeoff considering that it ensures that no CNS signals are contained in the EMG signal. The mixing matrix is constructed from jaw muscle recordings so that the spatial pattern is also as similar as possible to a real-jaw muscle recording. To ensure the availability of an EOG artifact component free of CNS signals, 20 blinks from channel Fp1 (see Figure 2 for electrode location) of an artifact recording were extracted, averaged, and then added to a zero baseline signal with varying gaps and a random multiplication factor ranging from 0.5 to 1.5.

Let W be the unmixing matrix obtained using BSS/ICA, and the mixing matrix A its inverse. A mixing matrix A was created for each of the two artifact signals $y_{\text{art}}$ by averaging over the mixing matrices calculated by the four algorithms for each artifact type. Thus, the mixing matrix for the EMG artifact was created by averaging mixing matrices calculated using BSS/ICA for jaw muscle artifacts and the mixing matrix for the EOG artifact by averaging mixing matrices calculated using BSS/ICA for eye blinks. The artifact signals $y_{\text{art}}$ were multiplied with the corresponding column i of these matrices and then added back to the background data $B_{\text{orig}}$, which yields the mixed signal $B^{\prime }$:

(1) $$B^{\prime }=B_{\text{orig}}+A_{i}y_{\text{art}}.$$

After re-extraction, the component $y_{\text{extr}}$, showing the maximum correlation with the introduced signal, was assumed to be the introduced artifact signal. This component was then removed from $B^{\prime }$ to obtain $B_{\text{clean}}$. The correlation between $B_{\text{org}}$ and $B_{\text{clean}}$ as well as $y_{\text{art}}$ and $y_{\text{extr}}$ was calculated as performance measure. For space reasons and because both measures allowed the same conclusions, only the results for the correlation between $y_{\text{art}}$ and $y_{\text{extr}}$ are shown in detail. The background signal had a length of 16714 samples recorded with the settings shown in Table 1. A sliding window was moved over the data with a length of 480 and an overlap of 240 samples. Each of these 480 sample segments was tested with the above procedure.

AMUSE and JADE were run with default parameters. Infomax was run with a limit of 32, 64, and 128 iterations to restrict runtime to less than two seconds and to analyze the influence of the number of iterations on runtime and quality. The weight matrix computed in the previous step was passed to the algorithm as an initial guess. A learning rate of 0.001 was used. FastICA was run with a maximum number of iterations of 100, 200, and 400. “Approach” was set to “symmetric,” as described in [25]. This causes the program to calculate all components in parallel, which is faster than a serial calculation if all components are to be estimated. The hyperbolic tangent function was chosen as “nonlinearity.” “Stabilization” was turned on, which prevents the program from getting stuck at certain points by halving the step size μ. Again the previous weight matrix was used as an initial guess. Without a restriction on the number of iterations, Infomax and FastICA tended to have a very high variance in computation time to convergence.

#### 2.1.2. BSS evaluation results

The performance of Infomax, JADE, AMUSE, and FastICA, determined using the evaluation method presented in the previous section, is shown here. The parameters stated in Section 2.1.1 were used.

**Figure 1:**
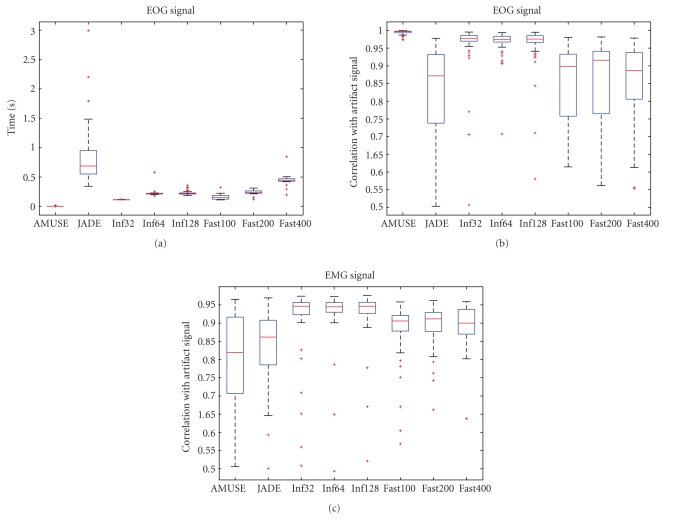
Boxplots showing times needed for extraction and EOG/EMG artifact extraction performance.

Runtimes are shown only for the EOG signal because they did not differ for the two artifact types (see Figure 1). AMUSE has a runtime lower by a factor of 50 compared to the second fastest algorithm, which is Infomax (with 32 iterations). JADE is the slowest algorithm and also exhibits the greatest variance. This excludes it from any application in online environments where a reliable runtime is vital to achieve optimal performance. The extraction of the artificially generated EOG artifact is also performed best by the AMUSE algorithm and worst by the JADE algorithm. Infomax does not perform as well as AMUSE, but it is clearly better than FastICA.

The performance of AMUSE drops for the extraction of the EMG signal. Infomax performs best with this type of artifact.

The data shows that no algorithm is optimal for both types of artifacts. Since the runtime of AMUSE is lower by a factor of 50, it is possible to employ both algorithms. This has the additional advantage that only AMUSE will run if EOG artifacts only are to be removed. Therefore, we decided to use AMUSE in combination with Infomax. This is realized by creating an R (4) for each of the algorithms. These matrices are then combined by multiplication and applied to the data.

### 2.2. Support vector machine training

#### 2.2.1. Training data

The artifacts recorded for SVM training and how the training of the SVMs was conducted are discussed in this section.

Four kinds of artifacts (jaw muscles, forehead movement, eye blinks, and eye movement), each recorded with four different subjects, were recorded to train the SVMs to classify various EEG artifacts. All recordings were made with the settings shown in Table 1.

The instructions to generate artifacts were presented to the subjects in random order, 40 times for each artifact type. Each presentation lasted 3 seconds. In total, this resulted in 19200 samples per artifact. These recordings were then split into blocks of 480 samples (i.e., 3 seconds), which were used to train the SVMs (at lengths lower than 300 samples, the classification rates began to deteriorate probably due to the fact that this is not sufficient for the BSS/ICA algorithms to perform separation on). This resulted in a training dataset with 2560 components per artifact. Every component was labeled by hand as either “artifact” or “nonartifact” by an expert. ICs containing EOG artifacts were mostly unambiguous. EMG artifacts tended to contaminate most ICs with varying degrees of intensity. Labeling every IC with only traces of EMG as artifact would result in too much of the nonartifact EEG data being removed. Therefore, only those ICs with strong EMG or no EEG were labeled as artifacts. The SVMs were all trained with an equal number of artifacts and nonartifacts (which were randomly selected from the set of available nonartifacts).

An RBF kernel [26] was used to classify the data based on their power spectral density (PSD) and the topography of the ICs (based on elements of the mixing matrix calculated with BSS/ICA). The PSD was calculated using Welch’s method [27] and split into 16 frequency bins ranging from 1.6 Hz to 80 Hz. The corresponding columns in the mixing matrix were used as the remaining 16 elements. This results in a feature vector which is the concatenation of spectrum and topography.

**Figure 2:**
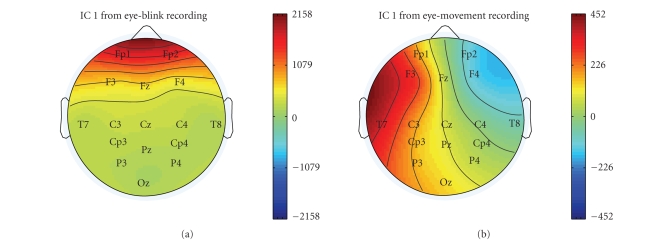
Topographic plots illustrating the differences in the features used for classification. The topographies of two ICs containing eye blinks (left) and eye movement (right) are shown.

**Figure 3:**
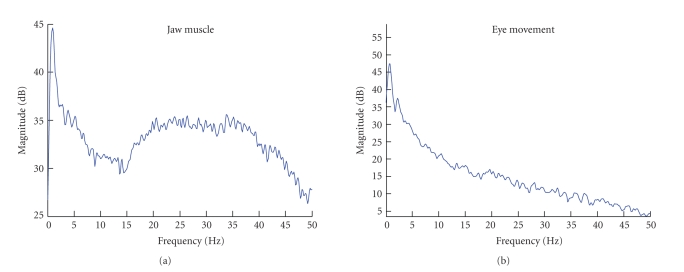
Power spectra showing the differences in the features used for classification, in this case of an IC containing jaw muscle contraction (a) and eye movement (b).

Figure 2 shows the difference between the topography of eye blinking and horizontal eye movement ICs. The eye blinking artifacts project most strongly on the frontal electrodes Fp1 and Fp2, whereas horizontal eye movement artifacts have a very distinct projection, in which electrodes on different hemispheres have a different polarity. The topography of EMG artifacts depends strongly on the muscles used, but they all show a characteristic power spectrum. An EMG power spectrum of a jaw muscle artifact is shown next to an eye movement power spectrum in Figure 3.

#### 2.2.2. Support vector machine classification performance

Classification rates of the SVMs for EOG and EMG artifacts using the features discussed in the previous section are briefly presented in the following section. The most apparent difference between EOG and EMG artifacts is that EMG artifacts contaminate a much higher percentage of the ICs than EOG artifacts (see Table 2). This is due to the fact that EMG artifacts are composed of multiple sources. These sources tend to be isolated into individual ICs, which leaves fewer components for the signal of interest. Additionally, traces of muscle activity remain in most of the components, varying in their intensity. That means that there is no hyperplane that clearly separates EMG and non-EMG components. This is reflected in the percentage of contaminated ICs and the percentage of correctly classified ICs during 20-fold cross validation (CV). Nevertheless, all classification rates lie above 90%, even above 99% in the case of EOG artifacts.

### 2.3. Implementation

The artifact filter is implemented using Matlab and integrated into the BCI2000 [23] software using an available Matlab interface. It consists of two major components. The first uses the signal data contained in buffer $Z_{\text{ICA}}$ and applies BSS/ICA to calculate the unmixing matrix W. Application of W to $Z_{\text{ICA}}$ yields the corresponding sources S:

(2) $$S={WZ}_{\text{ICA}}.$$

Then the SVMs are used to classify the sources S. We use the probability estimates p (values between zero and one, i.e., the probability that a component is not an artifact) of the SVM instead of classification results (zero or one) to construct a removal matrix. Probability estimates are calculated according to [30]. This avoids oscillation between removal and retainment in case of ambiguous components. Especially muscle artifacts are not cleanly isolated into single components, and those which contain only weak contamination might suffer from this problem when binary classification is used. p is used to construct a diagonal matrix D using the output of the SVMs for each of the four artifact types:

(3) $$\begin{array}{ll} D_{ii} & =p_{i},\;i=1,\ldots ,N,\\ D_{ij} & =0,\;\forall \;i\neq j,i=1,...,N,\;j=1,\ldots ,N, \end{array}$$

with N being the number of ICs.

D is combined with the unmixing matrix W and the mixing matrix A, as shown in (4), to create the removal matrix R that is applied to the current output sample $\sigma_{i}$ from buffer $Z_{\text{sig}}$ in the second component of the artifact filter (5) which yields a sample $\sigma_{i}^{\prime }$ reconstructed out of BSS/ICA components weighted according to the probability estimates p of the SVM:

(4) $$\begin{array}{ll} R & =ADW \end{array},$$

(5) $$\begin{array}{ll} \sigma_{i}^{\prime } & =R\sigma_{i}. \end{array}$$

The matrix R must be applied to every sample that passes through the filter. In a sequential approach in which the calculation of R takes $t_{R}>0$ this will prevent continuous output in online scenarios. Therefore the calculation of R and the application of R to the current output sample $\sigma_{i}$ must be performed in separate threads.

R must include mixing and unmixing matrices from AMUSE and Infomax. D must be calculated for each of the four SVMs (eye movement, eye blinks, jaw muscle and forehead muscle artifacts). All matrices are then combined by multiplication as shown in (6) and applied to the data.

(6) $$\begin{array}{ll} R= & A_{\text{AMUSE}}D_{\text{EOG}1}D_{\text{EOG}2}W_{\text{AMUSE}}\\ & \begin{array}{c} \times \end{array}\begin{array}{c} A_{\text{Infomax}}D_{\text{EMG}1}D_{\text{EMG}2}W_{\text{Infomax}}. \end{array} \end{array}$$

Additionally, it must be taken into account that the calculation of the unmixing matrix W using the BSS/ICA algorithm is based on more than one sample. Therefore, the data that are used for the calculation, that is, $Z_{\text{ICA}}$, must be representative of the sample $\sigma_{i}$ that the removal matrix R is applied to. If simply the last n samples of the data are used, newly occurring artifacts might not have a strong enough impact on the SVM to be classified as such, which would lead to the artifact not being removed from $\sigma_{i}$. Conversely, artifacts in the buffer which contaminate most of the samples, but not the newest sample, will cause the SVM to classify a component of $\sigma_{i}$ as an artifact which is in fact artifact-free. Hence, the incoming samples have to be delayed by half of the number of samples $k_{Z_{\text{ICA}}}$ in buffer $Z_{\text{ICA}}$ that contains the data for the BSS/ICA. This does not mean that a new sample arrives only every $k_{Z_{\text{ICA}}}/2$ samples but that the whole recording is shifted by this amount and the samples still arrive with the same interval.

It is inevitable that the same matrix is used for several samples as the time $t_{R}$, needed for the calculation of R, is greater than the time represented by one sample of the data.

## 3. RESULTS

The artifact filter is applied offline to three BCI datasets to evaluate the effect on determination coefficient plots and subject performance. It is shown that application of the filter increases performance in cases where artifacts randomly interfere with the control signal and decreases performance when artifacts are used to control the BCI. Additionally, the online functionality of the filter is discussed.

### 3.1. Offline analysis of BCI data

**Figure 4:**
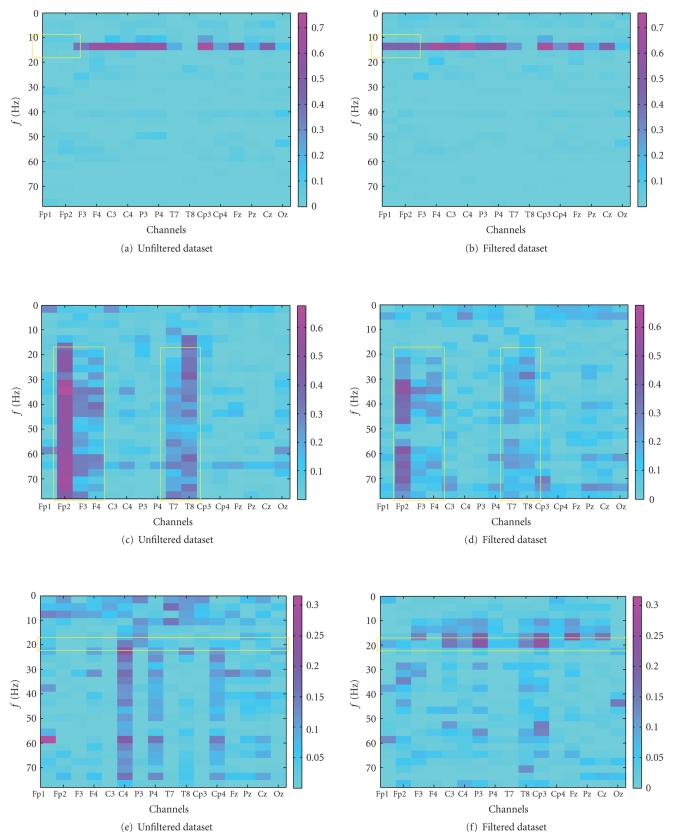
Determination coefficient ($r^{2}$) plots showing the correlation with a given task before (left) and after (right) filtering the signal. The first pair of plots (a) and (b) shows the effect of removing eye blinks uncorrelated with the task. The second pair (c) and (d) shows the removal of correlated muscle activity. The third pair (e) and (f) shows the effect of removing uncorrelated muscle activity. Regions of interest are marked with yellow boxes.

The data analyzed originate from μ-rhythm (i.e., a BCI controlled using imagined movement/planning) training sessions recorded with the settings shown in Table 1 during a BCI evaluation project [31] (none of the datasets was used during the training of the SVMs). The control a user has over the BCI can be evaluated with determination coefficient (Pearson product-moment correlation coefficient, $r^{2}$) values as those plotted in Figure 4. A higher value indicates modulation of the signal in a certain channel at a certain frequency in correspondence with the required task. In this case, the modulation of the signal was used to control the movement of a cursor to either the top or the bottom of the screen. Artifacts either increase or decrease the determination coefficient value at certain frequency ranges. If the artifact correlates with the task (mostly due to the attempt to control the BCI with muscle activity or an involuntary muscle contraction while trying to perform the mental task), it will increase the value, and if it does not (due to involuntary muscle spasms), it will decrease the determination coefficient value. In the former case, removal can be expected to decrease performance; in the latter, an increased or unchanged performance can be expected. This is what is intended since using artifacts to control a BCI violates the definition of it as being a nonmuscular communication channel. Plots 4(a) and 4(b) in Figure 4 show the effect of EOG artifact removal on data, which were recorded from a healthy subject presented 23 targets over 120 seconds, 22 of which were correctly chosen. Both of the frontal channels, Fp1 and Fp2, were contaminated with eye blinks. Since the blinks are uncorrelated with target location, this will have a negative influence on the determination coefficient value. As expected, the right plot, which shows the determination coefficient plot of the filtered data, has a visibly increased determination coefficient value on the frontal channels. On Fp1 the $r^{2}$ value increased from 0.0772 to 0.4539, and on Fp2 from 0.0929 to 0.4775. The maximum $r^{2}$ value increases from 0.7006 to 0.7585. This increase occurs in the same frequency range as the horizontal band of high determination coefficient values which existed previously, which is an indication of identical source (see box in 4(a) and 4(b)). Such a horizontal pattern is typical of successful μ-rhythm modulation. Because the control signal was not influenced significantly on the other electrodes, no increase in hit rate occurred.

In contrast to the top two plots, plots 4(c) and 4(d) show data from a subject that used muscle artifacts to control the BCI. This is reflected in the plot by the vertical structures (which indicate signals with a broad spectrum) that correlate with the task (which indicates that these signals were generated intentionally). Such patterns are visible on channels 2, 3, 4, 9, and 10 (see box in 4(c) and 4(d)). In total, 34 targets were presented, 29 of which were hit (85%). The filter does not entirely remove the EMG artifacts due to the previously described problem of muscle artifacts contaminating too many ICs. Nevertheless, the effect of the artifacts is significantly reduced, in particular below 20 Hz. Additionally, the absolute amplitude of the correlation of the EMG signals with the task was decreased visibly by the application of the filter (the maximum $r^{2}$ value decreases from 0.6777 to 0.5208). The removal led to a decrease of the simulated hit rate from 85% to 73%. The final case that is presented (see Figures 4(e) and 4(f)) also shows some contamination by EMG artifacts, even though not as strongly as the previous data. The subject was presented 34 targets, 17 of which were hit resulting in a hit-rate of 50%. The interesting fact about this dataset is that the removal of the EMG artifact reveals a horizontal structure in the range of 20 Hz (see box 4(e) and 4(f)). This indicates that there was some brain activity that was superimposed by EMG and could therefore not be used to control the BCI. The strongest increase of the determination coefficient value is based around electrode CP3 (channel 11), supporting this claim. On this channel, the maximum $r^{2}$ increases from 0.1204 to 0.2598. Additionally, this electrode was determined to be the most discriminative in initial screening sessions with the subject. The simulated performance of the subject increased from 50% to 74%.

### 3.2. Online functionality

The performance of the filter was tested by applying it to raw data and classifying it again. The data obtained are identical to the data that would have been obtained if the filter had been used in an online recording with the same settings. Of course, an arbitrary amount of time is available for the artifact filter in an offline setting. Therefore, the quality of artifact removal is potentially higher. Thus, the interval at which the removal matrix is updated was set to 5 sample blocks, which would be realistic in an online environment. Since the data was recorded with 8 samples per block at 160 Hz, this allows for 250 milliseconds per update.

The application of the removal matrix takes about 2.6 milliseconds per sample block. The calculation of a new removal matrix takes 145 milliseconds using both AMUSE and Infomax on the basis of 480 samples^1^. This is fast enough to update the removal matrix every five sample blocks with the above settings.

Using the BCI2000, several P300 recordings were made to ensure that running the filter would not have a negative impact on performance.

## 4. DISCUSSION

A filter that removes artifacts from EEG signals used in BCI systems was described. The data presented in this paper shows that the implementation of an online artifact filter using blind source separation and support vector machines is possible. This is achieved by delaying output by a constant amount between one and two seconds and by calculating and applying the removal matrix in two separate threads. Infomax was found to be the best ICA method to decompose EEG signals contaminated with myographic artifacts, but it had problems producing components which were completely free of contamination (especially if the myographic artifacts were very strong, as jaw muscle contraction). The second-order statistics-based algorithm AMUSE produced a very clean decomposition of recordings containing ocular artifacts, both eye movements and blinking. In turn, it was possible to train SVMs to recognize these artifacts with very high accuracy, because no contamination remained in the other components, making it easier to determine a separating hyperplane. Correspondingly, muscle artifacts could not be classified with such a high accuracy. The application of our artifact filter to BCI data demonstrates the usefulness of the technique. Performance increases were achieved in the case of uncorrelated muscle and ocular activity task. Additionally, a decrease in performance was apparent if the filter was applied to data in which the subject used muscle artifacts to control the BCI. Unfortunately, the removal was not complete, allowing some control of muscles of the BCI to remain. In future experiments, this might be improved by using more EEG channels which increase the number of ICs and therefore the chance that an EMG subspace can be isolated from the EEG subspace. Additionally, the method could be adapted for use with alternative BSS methods which are particularly suited to isolate EMG artifacts, for example, the canonical correlation analysis (CCA) method demonstrated in [32]. The CCA would be used instead of one of the ICA algorithms employed in this paper and be automated by using the available SVMs.

The feasibility of using ICA as a preprocessing technique for artifact detection has been shown in [33]. Performance increased for all presented artifact detection types after ICA preprocessing. It was noted though, that no performance increase was found when applying ICA to data contaminated with muscle artifacts. This is another indication that an alternative BSS or ICA algorithm should be used before classifying the data with SVMs.

Automated artifact removal techniques have been presented previously. For example, an automated regression analysis-based EOG removal method was presented in [34]. While being easy to apply it still suffers from the drawbacks of EEG-contaminated EOG channels (even though this was addressed by mounting the EOG electrodes in the proximity of the eyes and only calculating the regression coefficients from large EOG artifacts). Moreover, it is not possible to extend such a method to remove EMG artifacts.

Artifact isolation and removal using BSS/ICA algorithms automated by combination with SVMs or some other automatic classifier were already shown in [35]. While the applicability of the method presented in [35] is also restricted to ocular artifacts, we demonstrated a method based on the combination of BSS/ICA algorithms and SVMs that uses artifact features (topography and spectrum) that are available for all artifact types. Furthermore, we presented a design for online settings. A comparable method suitable for online use presented in [36], also not applied to EMG artifacts, depends on static models of artifact and brain signal topographies that do not adapt if the artifact changes, as the unmixing matrix calculated with BSS/ICA does. The method presented in [36] offers the advantage of having no delay and a reduced computational complexity.

We demonstrated that it is possible to implement an online-automated artifact removal technique on the basis of BSS/ICA and SVMs and illustrated the ameliorating effect on BCI performance.

## Figures and Tables

**Table 1:** EEG recording parameters.

	EEG recording parameters
Amplifier	16 channel biosignal (gtec, Graz, Austria)
Sampling frequency	160 Hz
Highpass filter	0.01 Hz
Lowpass filter	70 Hz
Notch filter	50 Hz
Electrode placements	16 channel subset of 10–20 systems (see Figure 2) [22]
Ground	Left mastoid (A1)
Reference	Right mastoid (A2)
Electrode material	Ag/AgCl
Recording software	BCI2000 [23]

**Table 2:** SVM training summary showing the percentage of independent components (ICs) that are contaminated by artifacts in the particular artifact dataset and the percentage of ICs classified correctly as artifact and nonartifact when using 20-fold crossvalidation (CV). Additionally, channel capacity calculated using the Blahut-Arimoto algorithm [28, 29] and the parameters C (error penalty) and γ (kernel parameter of RBF kernel) used to train the SVMs are shown.

Artifact	% ICs	% correct (CV)	Channel capacity	C /γ
Eye blink	6.40	99.39	0.8141	2000/0.0005
Eye movement	5.15	99.62	0.9373	2/0.5
Jaw muscle	52.34	92.26	0.6308	8/0.5
Forehead	19.34	91.51	0.6043	2/0.5

## Footnotes

^1^ Using an AMD Athlon X2 4400+ with 2 GB RAM.
